# In Leaders We Trust, or Should We? Supervisors’ Dark Triad Personality Traits and Ratings of Team Performance and Innovation

**DOI:** 10.3389/fpsyg.2021.650172

**Published:** 2021-06-14

**Authors:** Oana C. Fodor, Petru L. Curşeu, Nicoleta Meslec

**Affiliations:** ^1^Department of Psychology, “Babeş-Bolyai” University, Cluj-Napoca, Romania; ^2^Department of Organization, Open University of the Netherlands, Heerlen, Netherlands; ^3^Department of Organization Studies, Tilburg University, Tilburg, Netherlands

**Keywords:** supervisor dark triad personality traits, team performance, team innovation, collective narcissism, leader-member exchange

## Abstract

Our study tests in a sample of 87 organizational groups (297 employees and 87 supervisors) the mediating role of leader-member exchange (LMX) and collective narcissism in the relationship between supervisors’ dark triad (SDT) personality traits and ratings of team outcomes made by supervisors and team members. We show that LMX mediates the association between SDT and team performance and innovation as rated by team members, while collective narcissism mediates the association between SDT and supervisory ratings of team innovation and team performance. Moreover, collective narcissism also mediates the association between SDT and team innovation as rated by team members. Results show that team-level performance appraisal is influenced by supervisory attributes and that the quality of relational exchanges and collective narcissism are plausible mechanisms explaining this association. The use of supervisory ratings of team outcomes in empirical research should also account for the supervisory attributes.

## Introduction

A key job requirement for managers is to evaluate the performance of the individuals and groups they supervise. Extant literature in applied psychology explored various sources of bias in supervisory ratings of (individual) employee performance from liking (interpersonal affective regard, Lefkowitz, 2000) to the personality attributes of the supervisors (Deluga, 1998). Little attention, however, is shown to the way in which the supervisors’ attributes impact on team performance ratings. It is important to understand the biases in supervisory ratings for groups especially because supervisory evaluations of group outcomes (either carried out as part of the formal performance appraisal system or performed informally during team meetings) can have important motivational effects on subsequent performance episodes that the team engages in. Good team performance (recognized as such by supervisors and team members) fosters collective efficacy (Jung and Sosik, 2003) and team work engagement (Costa et al., 2014). On the other hand, low performance outcomes or inaccurate team performance ratings made by supervisors might undermine members’ belief in the team’s capacity to accomplish its tasks in the future, as well as team members’ willingness to spend time and effort on future tasks. In turn, such detrimental effects on team motivational and regulatory processes are likely to impede team performance in subsequent performance episodes (Marks et al., 2001; Jung and Sosik, 2003). On the other hand, the literature to date also seems to favor that team outcome data is collected from supervisors rather than self-reported by team members (Hülsheger et al., 2009; Rico, 2013). It is therefore important to explore whether supervisory ratings of team performance are influenced by supervisors’ personality traits that are independent of the task performance.

Another important element to consider is that research on rating effects mostly focused on individual differences as drivers of evaluative tendencies (Heidemeier and Moser, 2009; Hansbrough et al., 2020) and, although contextual factors were explored as important contingencies of evaluative tendencies (Hansbrough et al., 2015), little attention was devoted to relational variables that are likely to impact on team outcomes ratings. The aim of our paper is therefore to explore how the dark triad personality traits of the supervisor (SDT) shape the relational dynamics within teams and ultimately influence the ratings of team outcomes (performance and innovation) done by supervisors and team members, respectively. The dark triad (DT) includes three toxic personality traits, namely narcissism, psychopathy, and Machiavellianism (Paulhus and Williams, 2002). They are linked with social exploitation tendencies (Jonason and Webster, 2011) and have major implications for a wide variety of organizational outcomes (LeBreton et al., 2018). Recent reviews on DT (LeBreton et al., 2018) have called for more research that explores the association between DT and organizational (including team) outcomes and the mechanisms explaining this association.

Our paper builds on two theoretical perspectives namely the social exchange perspective (Blau, 1968; Cropanzano and Mitchell, 2005) and the relational perspective on leadership (De Cremer, 2003; Morgeson et al., 2010) to argue that supervisors are important sources of social influence in groups that shape their relational dynamics. On the one hand, we answer the call for more research on the association between leader characteristics and the perceived leader-member exchange (LMX; Andersen et al., 2020) and we claim that SDT reduces the perceived quality of LMX, which in turn shapes the evaluative tendencies for both the leaders as well as their members with respect to team outcomes. More specifically, we argue that the social manipulation and exploitation tendencies as the underlying interpersonal tendencies associated with SDT reduce the quality of LMX and ultimately impact on the ratings of team outcomes (Palmer et al., 2020). On the other hand, we answer the call for more research on social contagion mechanisms that explain the association between leadership behavioral tendencies and emergent states in teams (Owens and Hekman, 2016; Chen et al., 2019). We build on a contagion model of leadership (Sy and Choi, 2013) to argue that SDT impacts on team performance and innovation ratings by promoting collective narcissism as an emergent state in groups.

To summarize, our paper aims to test an integrative model in which the association between SDT and ratings of team outcomes (i.e., performance and innovation) as performed by supervisors and team members, respectively, is mediated by LMX and collective narcissism.

### Literature Review and Hypotheses

#### Supervisors’ Attributes and Ratings of Team Outcomes

Appraising performance outcomes for individuals and organizational teams serves a variety of purposes such as assessing training needs, managing performance or making personnel decisions and a common practice is to rely on the ratings done by supervisors as well as self-ratings. The literature on individual performance appraisal is abundant and has emphasized a consistent difference between supervisory and self-ratings of individual performance such that self-ratings are more lenient (Heidemeier and Moser, 2009). Supervisory ratings, on the other hand, seem to be considered more reliable (Conway and Huffcutt, 1997), are better linked to performance as measured by external criteria (promotions and salary, etc.) (Beehr et al., 2001; Atkins and Wood, 2002) and, as such, seem to be favored in both organizational practice and research.

At the team level of analysis, extant studies have also called for an increased use of team outcome ratings (i.e., innovation and performance etc.) done by independent raters such as the team leaders or subject matter experts instead of those performed by the team members themselves (Hülsheger et al., 2009; Rico, 2013). The aim is to reduce biases typically associated with self-ratings such as social desirability and leniency effects driven by impression management or self-deception strategies (Podsakoff et al., 2003; Hülsheger et al., 2009).

Social cognitive models of performance ratings have focused on the information processing tendencies that shape the evaluation of work outcomes by supervisors or team members. For instance, Heidemeier and Moser (2009) argue for a three-stage process whereby the rater collects the cues for team performance and innovation from various sources (i.e., observation of work results, stereotypes, and feedback from others etc.), then selects and integrates these cues into an overall assessment of the target and, finally, communicates his/her judgment via a final rating. However, these social-cognitive models of performance ratings have also looked at the factors that may alter these assessments by affecting information processing at various stages. An important factor that influences the collection of team performance and team innovation cues refers to the rater’s characteristics. As such, in this paper we further focus on the way the SDT personality traits (i.e., namely narcissism, psychopathy, and Machiavellianism) shape their evaluative tendencies via different mechanisms (i.e., relational processes vs. team emergent outcomes) and finally impact the ratings of team performance and innovation.

Narcissism describes an individual tendency of presenting him/herself in an overly positive manner, with interpersonal control and dominance tendencies, and a strong sense of entitlement (Raskin and Hall, 1979). Psychopathy reflects a lack of empathy and concern for others and a relative lack of remorse for destructive behaviors targeted toward others (Paulhus and Williams, 2002). Machiavellianism describes the tendency to use deceptive behaviors in social interactions. Machiavellian people are self-interested, cynical, and manipulative (Jones and Paulhus, 2009; Smith and Webster, 2017). Meta-analytic evidence suggests that although related, the three dimensions of the DT show enough distinctiveness to warrant separate exploration (O’Boyle et al., 2012). However, because we refer to the global effect of the DT on social relations and evaluative tendencies, we will focus on the SDT as a global indicator of social exploitation tendencies (Jonason and Webster, 2011). Such exploitation tendencies are clearly a dysfunctional feature of social influence in leadership and we argue that such interpersonal tendencies impact on team outcomes ratings by influencing two relational dimensions. First the social exploitation tendencies associated with high SDT decrease the quality of leader-member exchanges and second through social contagion it generates self-enhancement tendencies within groups that ultimately shape the team outcomes ratings.

#### Supervisors’ Dark Triad and Team Outcomes: The Mediating Role of LMX

In line with the social exchange theory (Blau, 1968; Cropanzano and Mitchell, 2005), we argue that organizational teams are arenas in which transactions occur, whereby social actors exchange rewards and obligations based on interdependence and reciprocation norms. Some of the most consequential relations that develop via such transactions are the ones among team members and their leaders. Within high quality leader-member exchanges, the leader provides access to resources (i.e., budget and goods etc.), opportunities (i.e., high visibility projects and career recommendations etc.) and socioemotional rewards (i.e., status, approval, and admiration etc.), whereas team members respond with the extra effort they put on the job. In time, both parties develop a sense of loyalty and trust, affective commitment and mutual support and the exchange is judged to be fair (Cropanzano and Mitchell, 2005).

Although engaging in relationship sustaining strategies seems to be favorable to leaders and team members alike, factors such as personality traits or situational constraints may alter this preference. In this paper we focus on the former and explore the negative implications of the SDT on the average quality of the dyadic relations s/he establishes with the team members and team outcomes.

The DT received substantial attention in the literature and various reviews (Furnham et al., 2013) and meta-analyses (O’Boyle et al., 2012) summarized its deleterious influences on work related behaviors and outcomes such as counterproductive work behavior and performance. For instance, high levels of Machiavellianism are associated with reduced trust in others (Dahling et al., 2009), a preference for leadership and management practices that promote control over team members (Lewin and Stephens, 1994; Kiazad et al., 2010) and increased unethical behavior (Kish-Gephart et al., 2010). Individuals with high levels of narcissism believe that usual standards do not apply to them and are entitled to their colleagues’ efforts and recognition. They need validation of an enhanced self-image (Chatterjee and Pollock, 2017), often at the expense of others, such as when taking credit for their team members’ accomplishments. Narcissists are likely to engage in aggressive communication, arrogant behavior (Morf and Rhodewalt, 2001; Vazire and Funder, 2006), and toxic leadership (Schmidt, 2008). High levels of psychopathy are linked with a disregard for social norms, dishonesty and impulsivity. Professionals with high scores on the psychopathy scale were perceived as having a poor management style, a reduced capacity to act as team players, and as more likely to engage in self-serving behaviors (Babiak et al., 2010; Barelds et al., 2018).

In short, supervisors scoring high on DT have a tendency to exhibit manipulative agentic behaviors, use social influence strategies aimed at (mis)using others to serve their personal interests and are less concerned with meeting social requirements such as the norm of reciprocity. By relying on the social exchange perspective on the implications of SDT for workplace behavior (O’Boyle et al., 2012) we argue that such behaviors tend to disturb the social harmony and the balance of reciprocity in social exchanges at work and ultimately may lead to retaliation or defensive behaviors from employees (Baloch et al., 2017; Palmer et al., 2020). Similarly, in their cascading model of the dark triad personality, Palmer et al. (2020) argue that leaders’ DT decreases the quality of social exchanges with their subordinates and it triggers retaliatory and counterproductive behaviors ultimately reducing collective performance. In line with these arguments, we therefore expect that the SDT attributes decrease the average quality of LMX at the team level.

At the individual level of analysis, low LMX has been consistently associated with reduced task performance, reduced organizational citizenship behavior and increased counterproductive work behaviors on the employees’ side (see Martin et al., 2016, for a meta-analysis). The effects are robust, regardless of the use of objective versus subjective ratings of the outcomes and are explained via motivational decrements occurring in low quality LMX, reduced trust, empowerment and satisfaction on the employees’ side (Martin et al., 2016).

Moreover, in teams where the quality of LMXs is low on average, team outcomes are also likely to be negatively affected by the impaired relational dynamics within the team. In particular, Palmer et al. (2020) have argued that low LMX affected by high levels of SDT will further promote a climate of perceived injustice and decreased trust (Park et al., 2019). In turn, the negative affective climate of the group will harm team members’ ability to communicate and collaborate with each other during task completion, thus leading them to perceive that they underperform. Ultimately, this will negatively affect the ratings of team outcomes (performance and innovation).

Hence, we hypothesize:

*H1:* SDT has a negative association with LMX.
*H2:* LMX mediates the relationship between SDT personality traits and ratings of team outcomes: performance (H2a) and innovation (H2b) done by supervisors and team members.

#### Supervisors’ Dark Triad and Team Outcomes: The Mediating Role of Collective Narcissism

In their leader-activation member-propagation model of leadership, Sy and Choi (2013) argue that through social and emotional contagion leaders shape the emotional experiences and expressions of their members. They show that through mood convergence, the emotions expressed by leaders are mimicked and shared by group members and, in time, the emotional climate in the group tends to be similar with the emotions expressed by the leaders (Sy and Choi, 2013). We extend this model to the behavioral realm of groups and argue that supervisory interpersonal strategies and the underlying beliefs associated with SDT are mimicked by the group members generating group level emergent phenomena such as collective narcissism.

At the individual level of analysis, collective narcissism is an extension of individual narcissism at the social level of the self and it reflects a set of beliefs (and the associated behaviors) about the superiority of the ingroup, along with a difficulty in sustaining such a positive image (Golec de Zavala et al., 2009). Individuals scoring high on collective narcissism perceive the group they belong to as an extension of themselves and expect others to recognize the special importance and positive uniqueness of the group (Marchlewska et al., 2020). At the group level of analysis, we argue that team level collective narcissism is a property of the team that emerges out of interpersonal interactions and it reflects a shared belief among team members in the superiority of the in-group.

By building on the leader-activation member-propagation model of leadership (Sy and Choi, 2013), our contention is that the supervisors’ behaviors associated with increased levels of SDT are central for the emergence of team level collective narcissism. As supervisors occupy central and powerful positions in groups, their behavioral patterns are more visible to the team members. Extant research shows that people in high-power positions engage in more disinhibited behaviors (Keltner et al., 2003), are more likely to publicly display their attitudes and are less likely to engage in perspective taking (Guinote, 2007). At the other end, individuals in low-power positions (i.e., team members) are more vigilant and better at registering the behavioral patterns that individuals in high-power positions (i.e., supervisors) engage in Keltner et al. (2003). Moreover, team members are more likely to further mimic the supervisor’s behavior in a conscious or non-conscious pursuit of affiliation and status goals (Lakin and Chartrand, 2003; Lakin et al., 2003, 2008).

In short, supervisors’ manipulative interpersonal behaviors associated with SDT and their self-centered narcissistic tendencies will activate through contagion, similar tendencies in their team members, thus fostering the development of a shared and exaggerated positive image of the in-group. By doing so, team members are more likely to achieve social integration within the group, the supervisor’s acceptance and the typical advantages associated with such a status (Kelly and Barsade, 2001).

In turn, when a team has a high level of collective narcissism, its members will seek to protect the exaggerated positive image of the group by engaging in impression management strategies. For instance, they are likely to overestimate the contribution of the ingroup and to distance themselves from the behaviors and the accounts of deeds that could harm their image (Putnam et al., 2018; Cichocka and Cislak, 2020). This will most likely lead to an overly positive evaluation of team outcomes performed by supervisors. In addition, previous individual level research has documented that employees tend to overestimate their performance, arguing that self-ratings of performance outcomes may be inflated due to self-esteem effects (Harris and Schaubroeck, 1988; Brown et al., 2001).

All in all, we expect that collective narcissism mediates the relationship between SDT and the ratings of team outcomes (performance and innovation) as provided by both team members and supervisors. Based on the above-mentioned arguments, we hypothesize the following:

*H3:* SDT has a positive association with collective narcissism.
*H4:* Collective narcissism mediates the relationship between supervisors’ dark triad personality traits and ratings of team outcomes: performance (H4a) and innovation (H4b) done by supervisors and team members.

Given that performance and innovation ratings reflect relevant team outcomes, we follow up on a suggestion received during the review process and explore the extent to which the association between SDT and the team outcomes rating congruence is mediated by LMX and collective narcissism. It is not unreasonable to assume that the high quality of leader member exchanges is beneficial for the congruence of team outcomes ratings, while collective narcissism as a shared belief in the superiority of the group is likely to be detrimental for the congruence of team outcomes ratings. We could expect that the indirect association of SDT and team outcomes rating congruence is negative such that SDT has a negative association with LMX, which in turn increases the similarity of team outcomes ratings. Moreover, we would expect a negative indirect association between SDT and team outcomes rating congruence mediated by collective narcissism such that SDT has a positive association with collective narcissism, which in turn decreases the congruence of team outcomes ratings.

## Materials and Methods

### Sample and Procedure

Data was collected in a sample of 297 employees (176 women) with an average age of 28.44 years old organized in 87 organizational groups (average group size was 7.83 as reported by team leaders) and 87 team leaders (51 women) with an average age of 33.47 years old. We have invited teams from a variety of contexts such as the IT sector, healthcare, higher education, HR and training, consultancy, constructions and sales to participate in the study. The inclusion criteria referred to the following: (1) the teams had to be recognized as distinct entities by other members of the organization; (2) with a clear goal associated with the production of products and/or services; (3) and engaged in a certain degree of interdependence (i.e., goal completion requires them to share resources and interact). Nine master students worked as research assistants and collected data by contacting teams via the HR department or directly via their team leaders or managers and invited them to participate in a study on “team dynamics.”

Data collection was carried out online. The online survey included a briefing on the nature of the study and treatment of data and participants expressed their consent by further engaging with the content of the survey. Participation was voluntary and participants could withdraw at any moment by exiting the online survey. Participants did not report their names or other identifying information during data collection and, because the survey did not include questions with the potential to embarrass the participants and with no consequences for their employability, no other written consent was asked from participants (in line 8.05 of the APA). In order to ensure the matching of data (reported by the team members and reported by the team leader), each data collection operator was instructed to create separate but matching links to the online survey for each team and the corresponding team leader (e.g., one link for Team 1 accessed by all corresponding team members and a matching link for the Leader of Team 1; a different link for Team 2 and a matching link for the Leader of Team 2). The data collection operators further distributed the corresponding links to the teams and matching team leaders whom they had access to and agreed to enroll in the study. Finally, data from all data collection operators were centralized in a single data base.

Team members were asked to fill in a survey that included questions regarding LMX, collective narcissism and team outcomes (performance and innovation). Supervisory reports were collected using the same items for team outcomes (performance and innovation) as for the team members. Supervisors were also asked to fill in the SDT scale. For further analyses we have used the teams for which we had at least two raters (two of the team members filled in the survey completely) which led to a sample of 297 team members nested in 87 teams and 87 team leaders. From 87 teams only 85 had full data on all variables included in the models tested in the paper.

### Measures

The *Dark Triad* of supervisors was evaluated using the *Dirty Dozen* scale presented in Jonason and Webster (2011). Each of the three dimensions was evaluated with four items. For narcissism, an example item is “I tend to want others to admire me”, for psychopathy an item example is “I tend to lack remorse” and for Machiavellianism and item example is “I have used deceit or lied to get my way”. Supervisors provided their answers on the DT scale by rating to what extent they agree to each of the statements; they used a 1–5 scale (1 = *fully disagree* to 5 = *fully agree*). Studies on DT have used either the individual scores for the three traits, under the assumption that their correlates may differ (Paulhus and Williams, 2002) or used aggregated scores for the DT (Jonason et al., 2010). Our hypotheses refer to the overall score and, in order to check whether it was warranted to use such an aggregate score, we used a principal component analysis (PCA). The PCA revealed that a single dominant factor with an eigenvalue of 5.51 covered more than 45.9% of variance in scores and all factors loaded significantly (loads higher than 0.43) in this dominant factor. Moreover, Cronbach’s alpha for all the items was 0.88, naturally higher than the Cronbach’s alpha for each individual scale (for narcissism was 0.82, for psychopathy was 0.68, and for Machiavellianism was 0.87). In light of these results, we considered appropriate to use an overall score for the SDT in further analyses, therefore we used the Bartlett dominant factor score because this score is an accurate indicator of the true factor score in the SDT dimensions (DiStefano et al., 2009).

*Collective narcissism* was evaluated with a nine-item scale presented in Golec de Zavala et al. (2009) and a sample item is “My group deserves special treatment”. The answers provided by each group member were recorded on a five points scale (1 = *fully disagree* to 5 = *fully agree*).

*Leader-member exchange* (LMX) was evaluated with the seven-item scale (LMX-7) developed by Graen and Uhl-Bien (1995) and a sample item is “How well does your leader understand your job problems and needs?”. The answers provided by each group member were recorded on a five-point Likert scale (1 = *not at all* to 5 = *to a great extent*, anchors varied depending on the item content).

*Team innovation* was evaluated with a four-item scale used in Drach-Zahavy and Somech (2001) and modified from West and Wallace (1991). Team members as well as supervisors were asked to fill in the scale with the instruction to reflect on the extent to which during the past 6 months the team engaged in such behaviors as the ones illustrated in the items (e.g., “The team developed innovative ways of accomplishing work targets/objectives”). Answers were recorded on a five-point Likert scale (1 = *not at all* to 5 = *to a great extent*).

*Team performance* was evaluated by supervisors and team members using a five-item scale presented in Rousseau and Aube (2010), examples of items are “Achievement of performance goals by my team is…” and the answers were recorded on a five-point Likert scale (1 = v*ery low* to 5 = *very high*).

*Overall rating congruence* was evaluated using the D index, typically used in rating convergence to capture the extent to which ratings from different sources (leaders and group members) on different dimensions (performance and innovation) are congruent and reflect substantial consensus or agreement. D index was computed as the sum of squared difference across rating sources and rating dimensions and it was reversed in order to reflect congruence rather than diversity in ratings (Edwards, 1995).

Participants were also invited to provide demographic data (i.e., age, gender, and education etc.). With respect to the education level, high school was coded as 1, college was coded as 2, bachelor’s level was coded as 3, masters was coded as 4, whereas Ph.D. or MBA were coded as 5.

#### Reliabilities and Aggregation Statistics

For the scales filled in by the group members we estimated the Cronbach’s alpha both at the individual level as well as at the group level of analysis (aggregated scores for the items at the group level). For the team performance rating of group members Cronbach’s alpha was 0.85 (0.88 for the group level of analysis).

For the team innovation ratings of group members, Cronbach’s alpha was 0.94 (0.96 for the group level of analysis). The internal consistency of the LMX scale was 0.88 for the individual level of analysis and 0.94 for the group level of analysis. Finally, for the collective narcissism scale the internal consistency at the individual level was 0.81 and at the group level of analysis was 0.85. The overall higher reliabilities at the group level illustrate the fact that indeed the items used in these scales target group level constructs (the referent is the group rather than the individual). For the supervisory team performance ratings, Cronbach’s alpha was 0.71 indicating a sufficient internal consistency of the scale. For supervisory ratings of team innovation, Cronbach’s alpha was 0.84 indicating a good internal consistency of the scale.

For some of the scales the unit of observation were the individual group members, yet the constructs referred to team level variables (i.e., collective narcissism, average quality of LMX, team performance and innovation as assessed by team members) as did the referent in the wording of the items. Therefore, the individual scores needed to be aggregated at the group level of analysis, in line with the composition framework of emergence (Kozlowski and Klein, 2000). In line with the recommendations used in groups research (Bliese, 2000), we have first computed the within group agreement indices (Rwg, James et al., 1993) as well as the intraclass correlation coefficients [ICC(1) and ICC(2), Bliese, 2000], in order to see whether there is enough agreement to warrant aggregation. The formula for ICC(1) is

$$\text{ICC}\left(1\right)=\frac{MS_{b}-MS_{w}}{MS_{b}+\left(\left(N_{g}-1\right)MS_{w}\right)},$$

where: *MS*_*b*_ is mean square between subjects, *MS*_*w*_ is mean square within subjects and *N*_*g*_ is the arithmetic mean of group sizes. The formula for ICC(2) is

$$\text{ICC}\left(2\right)=\frac{N_{g}\times \text{ICC}\left(1\right)}{1+\left(\left(N_{g}-1\right)\text{ICC}\left(1\right)\right)}.$$

The Rwg average scores, their standard deviation and range as well as the ICC(1) and ICC(2) are presented in Table 1. The scores reported in Table 1 fully support the aggregation of individual scores into group level indicators: above 0.70 for Rwg (James et al., 1993), above 0.25 for ICC(1) indicating strong effects, and values between 0.40 and 0.75 for ICC(2) indicating adequate reliability (Fleiss, 1986; LeBreton and Senter, 2008).

**TABLE 1:** Aggregation statistics.

	ICC(1)	ICC(2)	Mean Rwg (*SD*)	Range Rwg
Collective narcissism (Mb)	0.30	0.60	0.97 (0.02)	[0.93; 1.00]
LMX (Mb)	0.36	0.66	0.95 (0.04)	[0.79; 1.00]
Team performance (Mb)	0.32	0.61	0.93 (0.05)	[0.81, 1.00]
Team innovation (Mb)	0.34	0.63	0.88 (0.10)	[0.62, 1.00]

*ICC, intraclass correlation coefficient; Rwg, within group agreement index; *SD*, standard deviation; LMX, leader-member exchange; Mb, evaluated by the members.*

## Results

Means, standard deviations and correlations between study variables are presented in Table 2, with the results for the whole sample (*N* = 87 teams) presented below the diagonal and the results for the sample with no missing data (*N* = 85) are presented above the diagonal. For the regression and mediation analyses we have used listwise deletion, therefore the results reported further are based on *N* = 85.

**TABLE 2:** Means, standard deviations, and correlations.

	*M*	*SD*	1	2	3	4	5	6	7	8	9	10	11	*M*	*SD*
(1) Team size (L)	7.84	5.65	–	0.30**	−0.46**	–0.18	0.00	−0.24*	−0.27*	0.01	0.07	–0.04	−0.24*	7.88	5.68
(2) Gender of the leader (L)	0.59	0.49	0.30**	–	–0.01	−0.26*	–0.04	0.11	–0.03	0.09	–0.03	0.13	−0.29**	0.59	0.50
(3) Age of the leader (L)	33.47	9.17	−0.46**	–0.001	–	0.17	–0.03	0.43**	0.23*	–0.12	–0.11	0.03	0.09	33.33	9.23
(4) Education of the leader (L)	3.16	1.12	–0.19	−0.27*	0.16	–	0.15	0.02	–0.10	–0.01	0.15	0.04	–0.17	3.18	1.10
(5) LMX (Mb)	3.89	0.55	0.02	–0.04	–0.05	0.12	–	0.07	0.00	0.13	0.58**	0.38**	−0.25*	3.90	0.54
(6) Collective narcissism (Mb)	4.30	0.73	−0.24*	0.12	0.42**	–0.01	0.08	–	0.31**	0.32**	0.04	0.41**	0.25*	4.29	0.74
(7) Team performance (L)	4.12	0.46	−0.25*	–0.04	0.20	–0.08	0.04	0.30**	–	0.24*	0.20	0.03	0.00	4.14	0.46
(8) Team innovation (L)	3.53	0.85	0.01	0.05	–0.14	0.06	0.13	0.27*	0.27*	–	0.15	0.52**	0.06	3.56	0.81
(9) Team performance (Mb)	4.06	0.45	0.08	–0.03	–0.12	0.14	0.59**	0.05	0.22*	0.16	–	0.33**	–0.21	4.07	0.45
(10) Team innovation (Mb)	3.45	0.71	–0.03	0.12	0.004	0.05	0.40**	0.40**	0.08	0.53**	0.35**	–	0.06	3.47	0.70
(11) Supervisor dark triad (L)	0	1	−0.24*	−0.29**	0.10	–0.16	−0.27*	0.24*	–0.02	0.04	−0.22*	0.04	–	0	1

*p < 0.05; **p < 0.01; for results below diagonal *N* = 87 and for results above the diagonal *N* = 85. *SD*, standard deviation; Gender of the leaders was coded as 0 = men and 1 = women; L, evaluated by the leader; Mb, evaluated by the members; LMX, leader-member exchange.

As shown in Table 2, the team performance estimates of supervisors and team members were positively and significantly correlated (*r* = 0.22, *p* = 0.038) and the correlation corrected for the unreliability of the scales was *r* = 0.24, thus showing a rather moderate association between the two team performance estimates. This score is aligned with the meta-analytic estimates of 0.22 for the overall correlation between self and supervisory ratings of individual performance (Heidemeier and Moser, 2009). In other words, our results reflect the same tendency of obtaining a rather weak evaluation consistency between supervisory and self-ratings of team performance. Team innovation estimates of supervisors and team members were also positively and significantly correlated (*r* = 0.52, *p* < 0.001) and the correlation corrected for attenuation was *r* = 0.59, showing a rather high consistency of the supervisory and team member ratings.

We used the Ordinary Least Squares (OLS) regression analyses with robust standard errors based on the HC3 heteroskedasticity-consistent approach (HC3, heteroskedasticity-consistent estimator) presented in Hayes and Cai (2007) to predict LMX and collective narcissism based on SDT. We also included team size, gender, age, and education level of the leader as predictors in our analysis. Moreover, we predicted team outcomes (team innovation and performance) evaluated by supervisors and team members using LMX and collective narcissism.

The results are presented in Table 3.

**TABLE 3:** Results of the regression analyses.

Variable	LMX	Collective narcissism	TP (L)	TI (L)	TP (Mb)	TI (Mb)
Constant	3.93***(0.42)	3.09***(0.45)	3.89***(0.45)	1.86 (1.10)	2.18***(0.60)	0.13 (0.82)
Group size (L)	−0.004(0.01)	−0.01(0.02)	−0.02^†^(0.01)	−0.01(0.02)	0.004 (0.01)	−0.004(0.02)
Leader gender (L)	−0.10(0.13)	0.34*(0.17)	−0.11(0.13)	0.12 (0.18)	−0.03(0.11)	0.22 (0.16)
Leader age (L)	−0.001(0.01)	0.03**(0.01)	0.002 (0.007)	−0.03^†^(0.02)	−0.01(0.01)	−0.01(0.01)
Leader education (L)	0.04 (0.07)	0.03 (0.07)	−0.09*(0.04)	0.03 (0.10)	0.03 (0.04)	0.04 (0.07)
SDT (L)	−0.15*(0.07)	0.20*(0.08)	−0.10(0.06)	0.02 (0.12)	−0.03(0.07)	0.08 (0.07)
LMX (Mb)			−0.04(0.08)	0.14 (0.20)	0.45***(0.13)	0.48***(0.13)
Collective narcissism (Mb)			0.20*(0.08)	0.48**(0.17)	0.05 (0.07)	0.38*(0.15)
*N*	85	85	85	85	85	85
Adjusted *R*^2^	0.03	0.22	0.44	0.44	0.30	0.58
*F* statistic	1.42	6.99***	3.40**	2.03^†^	2.79*	5.03***

Unstandardized regression coefficients are presented in the table with the robust standard errors in between brackets. *p < 0.05; **p < 0.01; ***p < 0.001; Gender of the leaders was coded as 0 = men and 1 = women. TP, team performance; TI, team innovation; L, evaluated by the leader; Mb evaluated by the members; LMX, leader-member exchange; SDT, supervisor dark triad.

Although women tended to report lower SDT scores than men (*r* = −0.29, *p* = 0.007, see Table 2) (in line with previous research, Jonason and Davis, 2018), groups supervised by women tended to report higher levels of collective narcissism than groups supervised by men as indicated in Table 3 (the effect of gender on collective narcissism is β = 0.23, *p* = 0.05). The age of the supervisor positively predicted collective narcissism, β = 0.35, *p* = 0.003. Finally, as indicated in Table 3, SDT negatively predicted LMX (β = −0.27, *p* = 0.03), thus supporting Hypothesis 1 and it positively predicted collective narcissism (β = 0.27, *p* = 0.01), therefore supporting Hypothesis 3.

In order to test the indirect effects, we used the PROCESS macros (Hayes, 2017) to estimate, based on a resampling procedure, the indirect effect of SDT on ratings of team outcomes via LMX and collective narcissism. This procedure is particularly suitable for our analyses due to the fact that it can accurately test mediation in small sample sizes and that it is not sensitive to assumptions of normal distribution.

The results showed that the indirect effect of SDT on team performance as evaluated by the members was negative and mediated by LMX [effect size = −0.07, *SE* = 0.03, 95% CI (−0.13; −0.01)], yet LMX did not mediate the effect of SDT on team performance as evaluated by the leader [effect size = 0.01, *SE* = 0.01, 95% CI (-0.02; 0.04)]. As such, Hypothesis 2a was only supported for team performance as evaluated by team members. The indirect effect of the SDT on team innovation as evaluated by the members was negative and mediated by LMX [effect size = −0.07, *SE* = 0.03, 95% CI (−0.15; −0.01)]. LMX, however, did not mediate the effect of SDT on team innovation as evaluated by the leader [effect size = −0.02, *SE* = 0.03, 95% CI (−0.09; 0.03)]. Hypothesis 2b claiming a mediating role of LMX in the relation among SDT and team innovation was only supported for the innovation ratings done by team members.

On the other hand, collective narcissism mediated the effect of SDT on team performance as evaluated by the leader [effect size = 0.05, *SE* = 0.03, 95% CI (0.01; 0.10)], but not the effect of SDT on team performance as evaluated by team members [effect size = 0.01, *SE* = 0.02, 95% CI (−0.02; 0.05)]. Moreover, the indirect effect of SDT on team innovation as evaluated by the leader was positive and mediated by collective narcissism [effect size = 0.11, *SE* = 0.06, 95% CI (0.02; 0.25)], as was the indirect effect of SDT on team innovation as evaluated by team members [effect size = 0.09, SE = 0.05, 95% CI (0.01; 0.21)]. We can therefore conclude that Hypothesis 4a was supported for the supervisory ratings of team performance, while Hypothesis 4b received empirical support for both supervisory and team members’ ratings of innovation.

**FIGURE 1:**
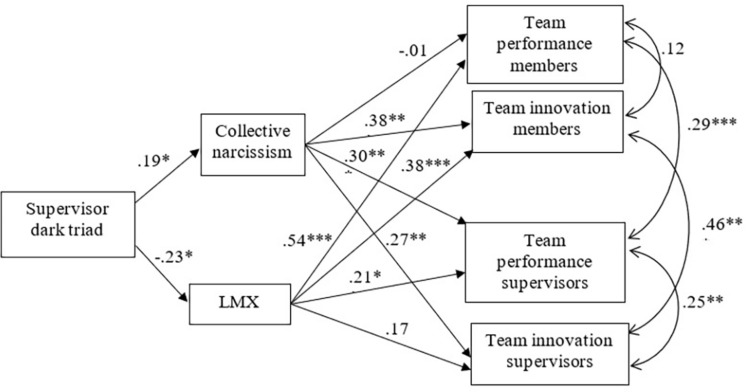
Results of overall model. Standardized path coefficients as presented in the final model (^∗∗∗^*p* < 0.001; ^∗∗^*p* < 0.01; ^∗^*p* < 0.05); Fit indices: χ^2^ = 10.83, d*f* = 7, *p* = 0.15, CMIN/d*f* = 1.55, CFI = 0.97, TLI = 0.90, RMSEA = 0.06; LMX, leader-member exchange.

In order to test the overall model stating that the influence of the SDT on different outcomes is mediated by LMX and collective narcissism we used Structural Equation Modeling (SEM) with the AMOS software (version 19). Using a maximum likelihood procedure, we estimated simultaneously the two mediators in relation to the four dependent variables and also allowed the error terms of the dependent variables to covariate (Tomarken and Waller, 2005). The results of this mediation analysis are presented in Figure 1. The overall chi square value (χ^2^ = 10.83, *df* = 7, *p* = 0.15) and the RMSEA = 0.06 (lower than 0.08 as indicated in Browne and Cudeck, 1993) scores indicated that the model fitted the data well. Moreover, the TLI index was 0.90, indicating the that model could not be substantially improved and the NFI = 0.94 and CFI = 0.97 also indicated a good model fit. The results of the SEM analysis supported the indirect effects found significant in the resampling procedure.

In order to test the additional exploratory mediation hypotheses, we used the PROCESS macro (Hayes, 2017) with the same control variables (team size, leader’s gender, age, and education). As dependent variable we used the rating congruence index that captures the general convergence between rations done by leaders and team members for team performance and team innovation. The indirect association between SDT and general rating congruence, mediated by LMX was negative and significant [effect size = −0.10, *SE* = 0.05, 95% CI (−0.20; −0.01)] as expected. However, the indirect association between SDT and general rating congruence as mediated by collective narcissism, although negative as expected, it was not significant [effect size = −0.05, *SE* = 0.04, 95% CI (−0.14; 0.01)]. We can therefore conclude that only LMX as a relational variable significantly mediated the association between SDT and overall rating congruence. Other relevant findings refer to a positive association between the education level of the leader and rating congruence (β = 0.40, *p* = 0.001) as well as between team size and rating congruence (β = 0.32, *p* = 0.003).

## Discussion

Our study answers the call for more research on the collective implications of SDT (O’Boyle et al., 2012; LeBreton et al., 2018) and on the mechanisms that explain the association between SDT and organizational outcomes (LeBreton et al., 2018). In short, the study explores the way SDT shapes the evaluations of team performance and innovation as rated by either team members or by supervisors. Moreover, we look into the role of LMX and team level collective narcissism as two mechanisms explaining the relation between SDT and team outcomes.

The most notable finding is that SDT influences the performance ratings made by the supervisors and by the team members through different mechanisms. LMX as assessed by team members explains the negative effect of SDT on ratings of team performance and innovation done by team members, but not by supervisors. Previous research showed that narcissistic leaders decrease the quality of information exchanges and ultimately group performance (Nevicka et al., 2011) and our research adds to this stream of research. This finding is in line with the social exchange perspective on the implications of SDT for workplace behavior (O’Boyle et al., 2012) and the cascading model of the dark triad personality (Palmer et al., 2020). These frameworks claim that the manipulative behaviors associated with the high levels of SDT disturb the social fabric of the group and the balance of reciprocity in social exchanges at work. As team members are at the losing end of these exchange relationships, the reduced quality of LMX will further impair team performance as assessed by team members, probably via stimulating their engagement in counterproductive and retaliatory behaviors in an attempt to restore the lost balance in reciprocity. In line with the social cognitive model of performance ratings (Heidemeier and Moser, 2009), we argue that this effect stands for team members as raters of team outcomes (and not supervisors) as the team members’ perception on (the impaired) LMX might direct their attention (and not their supervisors’) on selecting team performance cues that better reflect their current (dysfunctional) team dynamics.

Collective narcissism, on the other hand, explains the effect of SDT on ratings of team performance and innovation done by supervisors and on ratings of team innovation done by members. Supervisors displaying behaviors and beliefs congruent with high SDT (i.e., presenting oneself in a positive light, asserting dominance and entitlement etc.) are likely to contaminate the group such that team members also develop an exaggerated positive image of the in-group and, as such, achieve social integration and the leader’s acceptance. This finding is in line with the leader-activation member-propagation model of leadership (Sy and Choi, 2013) and brings initial empirical support for extending it from the dyadic to the team level of analysis. In turn, high levels of collective narcissism contribute to enhanced ratings of team performance and innovation done by supervisors as team members are likely to publicly engage in impression management strategies. Consequently, supervisors are likely to have access to an increased sample of positive performance cues when appraising team outcomes.

Another important insight concerns the positive correlation between collective narcissism and the age of the leader, plausibly explained by the fact that leader’s age is a proxy for experience, seniority and respect, therefore the group members may feel superior as a group as well. Indirectly such an argument ties to the tenure of the leader within the group, such that the leader’s longevity within the group fosters shared beliefs on the cohesiveness and superiority of the group. Moreover, groups led by women tend to report more collective narcissism than groups led by men. In line with the social role theory (Eagly and Johnson, 1990), women leaders are more oriented toward establishing and maintaining harmonious relations within the group and they have a democratic rather than autocratic leadership style. Therefore, the group members may perceive themselves as being more empowered and superior to other groups.

Concerning the overall rating agreement, only LMX was a significant mediator of the SDT to general rating congruence showing that, in general, the quality of the LMX is a critical factor for the convergence of the team member and leader ratings of team outcomes. The quality of LMX is also likely to generate a more accurate shared understanding on how the team performs. Future research could use objective indicators of team outcomes (if available) to compute the true accuracy of team outcomes rated by supervisors and team members. Two other factors had a significant association with general rating congruence, namely leader’s education and team size. The positive association between leader’s education and rating congruence is possibly explained by the fact that highly educated leaders are better equipped to accurately evaluate the performance of their subordinates. The positive association between team size and general rating congruence has two plausible explanations. First, it is possible that, in larger teams, intragroup differences in ratings reflect better the outcomes and converge toward more accurate ratings. Second, the better rating congruence in larger teams could be the result of a salience effect, as larger teams may have less controversial (global) performance indicators than smaller teams.

To summarize, our paper makes several important contributions to the literature. First, our study expands the growing body of literature on the negative aspects of leadership, namely the DT personality traits (LeBreton et al., 2018; Mackey et al., 2020). While the deleterious interpersonal nature of DT has been acknowledged, relatively few empirical studies explored the implications of these traits for team processes and outcomes (LeBreton et al., 2018; Palmer et al., 2020). Previous team level research mostly focused on the effects of team composition in terms of DT personality traits (i.e., average level of team members’ DT) and has shown that DT hurts team processes and emergent states (i.e., reduces cohesion and commitment) and impedes performance (Baysinger et al., 2014). We contribute to this stream of research and show that the exploitative, deceptive, and callous nature of supervisors with high levels of DT is also taxing for the team as such behaviors disturb the social fabric of the group. Our findings also partially align with Palmer et al.’s (2020) theoretical claims, arguing that the leaders’ DT personality traits (the CEO’s DT, in their case) may ultimately hurt firm performance via a propagation effect that relies on disturbed social exchanges within groups and retaliatory behaviors that cascade throughout the organization.

Second, our study contributes to the research on performance appraisal and extends insights on the biases in performance appraisal induced by supervisors’ attributes to the group level of analysis. The study shows that although supervisory ratings seem to be favored when assessing team outcomes (Hülsheger et al., 2009; Rico, 2013), the relational dynamics between the leaders and their teams deserves more attention when the group is the level of analysis and the referent of outcomes ratings. In particular, we join the call for more research that points to mechanisms that explain the association between SDT and organizational outcomes (LeBreton et al., 2018) and we simultaneously test two relational mediators linked to social exchange (i.e., LMX) and social contagion (i.e., collective narcissism) that explain the influence of SDT on ratings of team innovation and performance. Previous research has shown the association between leaders’ behaviors and LMX, as well as the beneficial effects of high quality LMX for positive individual behaviors and outcomes (Schuh et al., 2018; Andersen et al., 2020; Götz et al., 2020; Xie et al., 2020). Similarly, previous research has explored the way social contagion mechanisms explain the association between leadership behavioral tendencies (i.e., leader humility) and emergent states in teams (i.e., collective humility) (Owens and Hekman, 2016). We also add to these streams of research and show that reciprocity (as an underlying feature of social exchanges among leaders and their team members) and contagion may play out as distinct antecedents of performance ratings and are the linking pin with supervisors’ DT traits. LMX as assessed by team members explains the negative effect of SDT on ratings of team performance and innovation done by team members, but not by supervisors. Collective narcissism, on the other hand, explains the effect of SDT on ratings of team performance and innovation done by supervisors and on ratings of team innovation done by members.

### Practical Implications, Limitations and Future Research Directions

These results have important implications for using supervisory ratings of team performance. Within organizations, team supervisors often provide formal or informal performance ratings for the teams they lead. When such ratings are positive and accurate, they have important motivational and regulatory effects. The opposite occurs when team outcome ratings performed by supervisors are inaccurate. Our findings show that supervisory ratings of team performance and innovation are influenced by SDT personality traits and their deleterious influence on the relational processes within the group. One way to mitigate such effects might be to raise supervisors’ awareness on these effects as such interventions have proved to be beneficial (Winning Russo and Shoemaker, 2001). Moreover, organizations could rely at least partly on objective performance (e.g., number or errors in lines of code for a programming team etc.) and innovation criteria (e.g., number of patents or improvement ideas submitted by the team etc.) when assessing team outcomes.

In applied psychology, collecting performance ratings from supervisors is widely used in order to reduce concerns for common method bias (when other variables are evaluated using reports from team members). However, when using such ratings, controlling for individual differences likely to affect such ratings is useful. Moreover, team innovation ratings collected from team members could be used if controlled for collective narcissism. Future research could explore the association between objective indicators of team performance and the performance rated by members and supervisors while controlling for LMX and collective narcissism.

In our research we have argued that collective narcissism emerges from a contagion process. However, collective narcissism could also emerge as a result of external pressures and threats targeted toward the group (Golec de Zavala et al., 2009). It is not unreasonable to argue that the interpersonal manifestations of the SDT could also be perceived as threatening by the group members and actually collective narcissism will emerge as a collective defense mechanism to the SDT. Future research, could explore the extent to which collective narcissism emerges from leadership contagion or is a defense mechanism to SDT.

Another relevant insight that emerges from our results is that team performance ratings stemming from different sources are likely to be driven by different factors. Therefore testing “universal” models that explain team performance based on particular antecedents (e.g., input-process-output models) might be more challenging as the results may not be replicable for different rating sources. Such explanation is also supported by the higher correlation between innovation ratings of leaders and team members than between the performance ratings. A plausible explanation could be the multidimensional nature of group performance as a global indicator (leaders may have focused on other performance cues than group members did), while the innovation metric is less disputable and, as a consequence, it yields more consistent estimates. This result calls for a clear definition of performance dimensions, in order to be able to match and compare members with leader ratings in a meaningful way.

Finally, two other interesting findings concern the positive association between the leaders’ age (probably as a proxy for experience) and collective narcissism, and the fact that groups led by women leaders tend to report more collective narcissism than groups led by men, possibly explained by the more empowering and democratic orientation of women leaders. Future research could explore whether the positive association between age and collective narcissism is explained by leader tenure, as well as whether the relational orientation explains such gender differences in group leadership.

Next to the contributions, the study also has several limitations. First, the study is cross-sectional, therefore causal claims are not warranted. It is, however, unlikely that the reversed causation is plausible, as the personality traits of the supervisors are unlikely to be affected by LMX and collective narcissism. Second, although we have collected outcomes ratings from different sources, common method bias could be a concern for the mediation chains in which all variables ratings were collected from team members. This concern is likely illustrated by the higher correlations between LMX and collective narcissism on the one hand and team performance and innovation as rated by members than as rated by leaders. With respect to our overall mediation results, we believe that common method bias is less of concern as each of the mediation chains includes at least one variable evaluated from a different source. Although our study did not aim to fully disentangle the common source effects, our results show that different mechanisms explain outcome ratings from different sources. Such results call for using a more integrative approach to ratings of team outcomes and include both ratings from team members as ratings from supervisors.

## Conclusion

To summarize, our paper tested an integrative model exploring the mechanisms that explain the association between SDT and ratings of team outcomes as performed by supervisors and team members. We have argued that team leaders scoring high on SDT create a toxic relational environment in teams and based on social exchange theory we predicted that SDT decreases the quality of LMX while based on the contagion model of leadership we predicted that SDT fosters collective narcissism. These hypothesized main effects were fully supported by our analyses. We further argued that LMX and collective narcissism are mechanisms that explain the association between SDT and ratings of team outcomes. The mediation analyses reveled a more nuanced picture on the way in which the two mediators work for outcomes ratings made by team members and team leaders. Our results show that LMX mediates the association between SDT and team performance and innovation as rated by team members, while collective narcissism mediates the association between SDT and supervisory ratings of team innovation and team performance. In other words, the quality of social exchanges between team members and leaders matters most in the eyes of the team members when they rate team outcomes, while team members’ shared beliefs in the superiority of their group matters most in the eyes of their supervisors when they rate team outcomes. Finally, our results also show that collective narcissism mediates the association between SDT and team innovation as rated by team members.

## Data Availability Statement

The raw data supporting the conclusions of this article will be made available by the authors, without undue reservation.

## Ethics Statement

The studies involving human participants were reviewed and approved by Babeş Bolyai University Ethical Review Board. The patients/participants provided their written informed consent to participate in this study.

## Author Contributions

OF and PC were involved in study the design, data collection and analysis, and writing and editing the manuscript. NM was involved in study the design, data analysis, and writing and editing the manuscript. All authors contributed to the article and approved the submitted version.

## Conflict of Interest

The authors declare that the research was conducted in the absence of any commercial or financial relationships that could be construed as a potential conflict of interest.
